# Current Enhancement Behavior Under Positive Bias Stress in a-InGaZnO Thin-Film Transistors

**DOI:** 10.3390/mi17080928

**Published:** 2026-08-01

**Authors:** Guangan Yang, Xu Guo, Tianzhen Li, Zheng Guo, Geng Huang, Yan Jiang, Huabin Sun, Hong Zhu

**Affiliations:** 1 The College of Integrated Circuit Science and Engineering, Nanjing University of Posts and Telecommunications, Nanjing 210023, China; 2 The School of Microelectronics, Jiangsu Vocational College of Information Technology, Wuxi 214153, China

**Keywords:** amorphous InGaZnO (a-IGZO), thin-film transistors (TFTs), positive bias stress (PBS), interface charge, positive charges neutralization

## Abstract

Enhancement behavior on saturation current of output characteristics in amorphous indium–gallium–zinc oxide (a-IGZO) thin-film transistors under positive bias stress (PBS) is investigated. The threshold voltage (*V*_th_) of the a-IGZO TFT demonstrates a typical positive shift during PBS. Notably, the output current at an identical overdrive voltage (*V*_ov_) initially rises with increasing bias stress duration. The observed phenomena are elucidated by the detrapping of positively charged defects situated at the interface between the dielectric and active layer due to electrons induced by bias stress during PBS, which diminishes carrier scattering at the channel interface. Following the full release of positive interface charge, additional electron trapping states emerge. The presence of trapped electrons intensifies carrier scattering at the channel interface, leading to a degradation in drive current. Low-frequency noise (LFN) measurements are conducted to verify the suggested mechanism of PBS instability in a-IGZO TFTs. Moreover, the energy distribution of PBS-induced traps is shown by *C-V* characterization to be exponential, dominated by shallow traps. Passivation greatly enhanced the PBS stability, which is attributed to hydrogen doping-induced defect passivation and the shielding of the back-channel interface from the air atmosphere.

## 1. Introduction

Due to their exceptional device electrical properties, notable transparency, substantial large-area uniformity, and cost-effectiveness, amorphous InGaZnO (a-IGZO) thin-film transistors (TFTs) have found widespread commercial utilization in both indoor and outdoor displays, gradually replacing amorphous silicon TFTs (a-Si TFTs) especially in backplane applications of active-matrix displays (AMDs) such as AMOLED and AMLCD [1,2,3,4,5,6]. The application scope of a-IGZO TFTs extends to the promising realm of very large-scale integration circuits (VLSI) for the upcoming generations, owing to their low process temperature and compatibility with Si-based processes, which enable monolithic three-dimensional integration and flexible electronics [7,8,9,10,11]. However, for practical scenarios, the instability of a-IGZO TFTs presents a critical concern, especially in terms of threshold voltage (*V*_th_) variation under bias stress (e.g., positive bias stress (PBS) or negative bias stress (NBS)), leading to irregular pixel brightness or circuit malfunction [12]. Such instability is closely associated with charge trapping, defect creation, or environmental interactions. This is intrinsically linked to the amorphous nature of a-IGZO, where oxygen vacancies (V_O_) serve as the primary source of free carriers, a characteristic that is inherently a double-edged sword, as these metastable defects can be readily perturbed by the migration or incorporation of extrinsic mobile species (e.g., hydrogen, moisture, or ambient oxygen) induced by external bias and environmental exposure [13]. Therefore, achieving electrical stability is the only way that a-IGZO TFT can be commercialized.

Over the past decade, extensive research has focused on the positive bias stress (PBS) instabilities of a-IGZO TFTs [12,13,14,15,16,17,18,19,20,21,22]. Prevailing perspectives typically attribute the positive threshold voltage shift (Δ*V*_th_) to electron trapping at the dielectric/channel interface, alongside complex mechanisms such as V_O_ ionization and hydrogen migration. While researchers have rigorously investigated the impacts of fabrication processes, device structures, and environmental factors on Δ*V*_th_, the majority of these studies focus solely on the stability of the turn-on voltage, often overlooking the temporal evolution of the device’s actual driving performance [23,24,25,26,27,28]. In practical active-matrix display and circuit applications, the operational integrity of the transistor is defined not only by its *V*_th_ but also by its current-driving capability and carrier mobility (*μ*_FE_), both of which directly dictate the switching speed and signal processing fidelity of the integrated circuits. However, limited attention has been devoted to the complex “turnaround” behaviors of saturation current and mobility during PBS, which are crucial for a comprehensive understanding of degradation mechanisms. Although several studies have reported mobility enhancement in a-IGZO TFTs under specific conditions such as cryogenic operation [26], post-stress recovery [29], or structural engineering [30], these effects are typically induced by external stimuli or device modifications and exhibit monotonic behavior.

In this article, the influence of PBS on such electrical properties as V_th_, output current, and field-effect mobility (*μ*_FE_) of the a-IGZO TFTs were investigated in detail. *V*_th_ keeps shifting right throughout the bias stress duration. The output current is monitored at a fixed overdrive voltage (*V*_ov_). It is observed that the saturation current boosts first and then drops with the increasing stress time. Consequently, an enhancement in *μ*_FE_ is observed during the early stages of the PBS process. The turnaround phenomenon is attributed to the neutralization of positively charged defects, predominantly determined by V_O_ at the channel/dielectric interface, by bias-induced electrons during PBS. The reduction in interface charge density alleviates carrier scattering, supported by low-frequency noise (LFN) measurements. Additionally, *C-V* measurements demonstrate that the traps created by PBS follow an exponential energy distribution, in which shallow traps are dominant. The PBS stability was significantly improved after passivation, a result that is ascribed to the passivation of defects by hydrogen doping and the prevention of air exposure at the back-channel interface.

## 2. Materials and Methods

**Figure 1:**
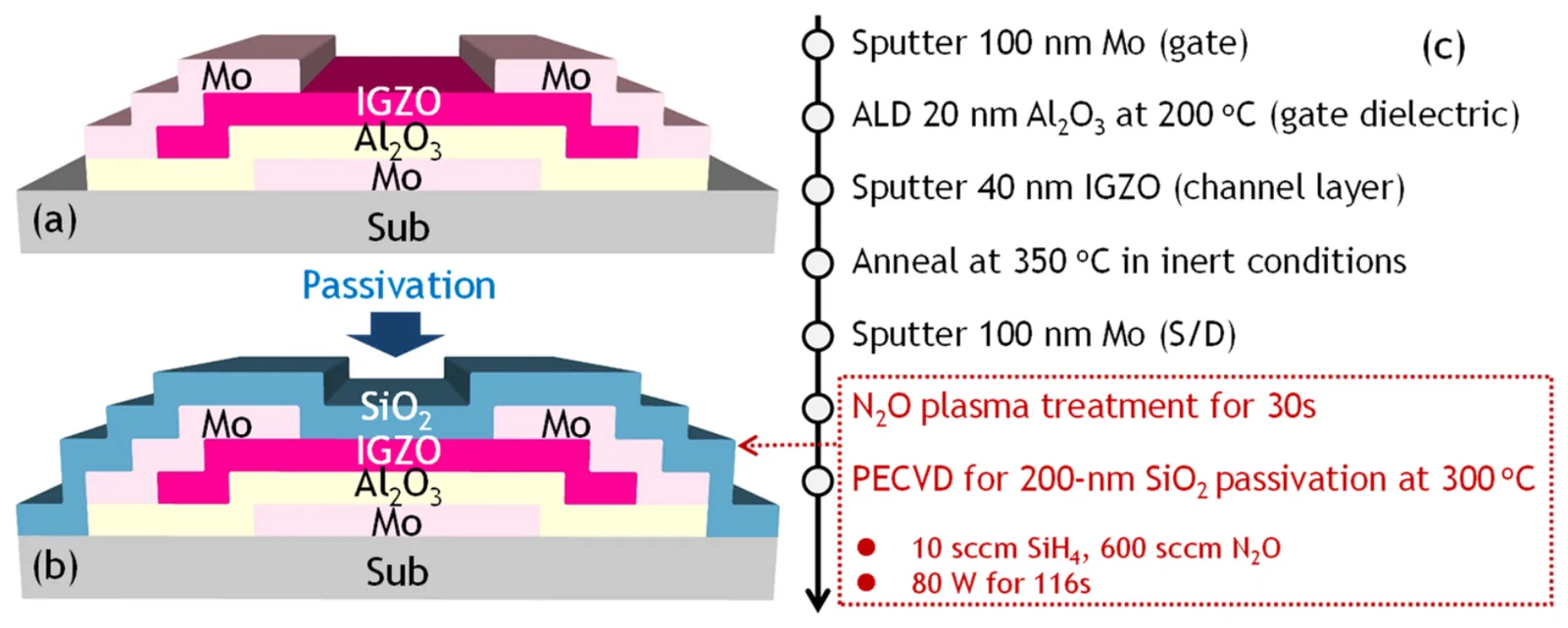
Schematic view of the a-IGZO TFT (**a**) without and (**b**) with passivation. (**c**) Fabrication process flow of the studied a-IGZO TFTs in this work.

Figure 1a presents the schematic view of the a-IGZO TFT without passivation used in this work, which is fabricated on a Si/SiO_2_ substrate. First, A 100 nm thick Mo layer was deposited via sputtering and patterned as the gate electrode, followed by the gate dielectric of 20 nm thick Al_2_O_3_ was globally deposited by the ALD with the substrate temperature of 250 °C. Next, a 40 nm thick a-IGZO layer (In_2_O_3_:Ga_2_O_3_:ZnO = 1:1:1 mol%) was formed by a magnetron sputtering with Ar/O_2_ ratios of 50/0.5 sccm, at the power of 60 W and working pressure of 5 mTorr. annealed under 350 °C in Ar atmosphere for 1 h. A 100 nm thick Mo was deposited and patterned as the source/drain (S/D) contact. Finally, a 200 nm thick SiO_2_ capping layer, formed by plasma-enhanced chemical vapor deposition (PECVD), serves as the passivation layer. The SiO_2_ passivation layer was deposited at a substrate temperature 300 °C, where the 300 °C is a moderate process temperature designed to maintain the structural and chemical integrity of the a-IGZO channel. At this temperature, the amorphous network of the IGZO thin-film remains thermally stable, and no significant degradation of the electronic properties is induced. The fabricated device with a bottom gate and staggered contacts has a channel width (*W*) and length (*L*) of 200 μm and 50 μm, respectively. The passivated a-IGZO TFT devices were also fabricated to investigate the influence of the PBS effect [Figure 1b]. A gate bias stress (*V*_stress_) of +5 V was applied to the a-IGZO TFT with source and drain grounded using an Agilent B1500 semiconductor parameter analyzer (Agilent Technologies, Santa Clara, United States). Before and after each PBS node, transfer curves at a drain voltage (*V*_d_) of 0.1 V and output curves at an overdrive voltage (*V*_ov_) of 2 V were acquired. Additionally, LFN measurements were executed to elucidate the degradation mechanism of the a-IGZO TFT under PBS.

## 3. Results and Discussion

**Figure 2:**
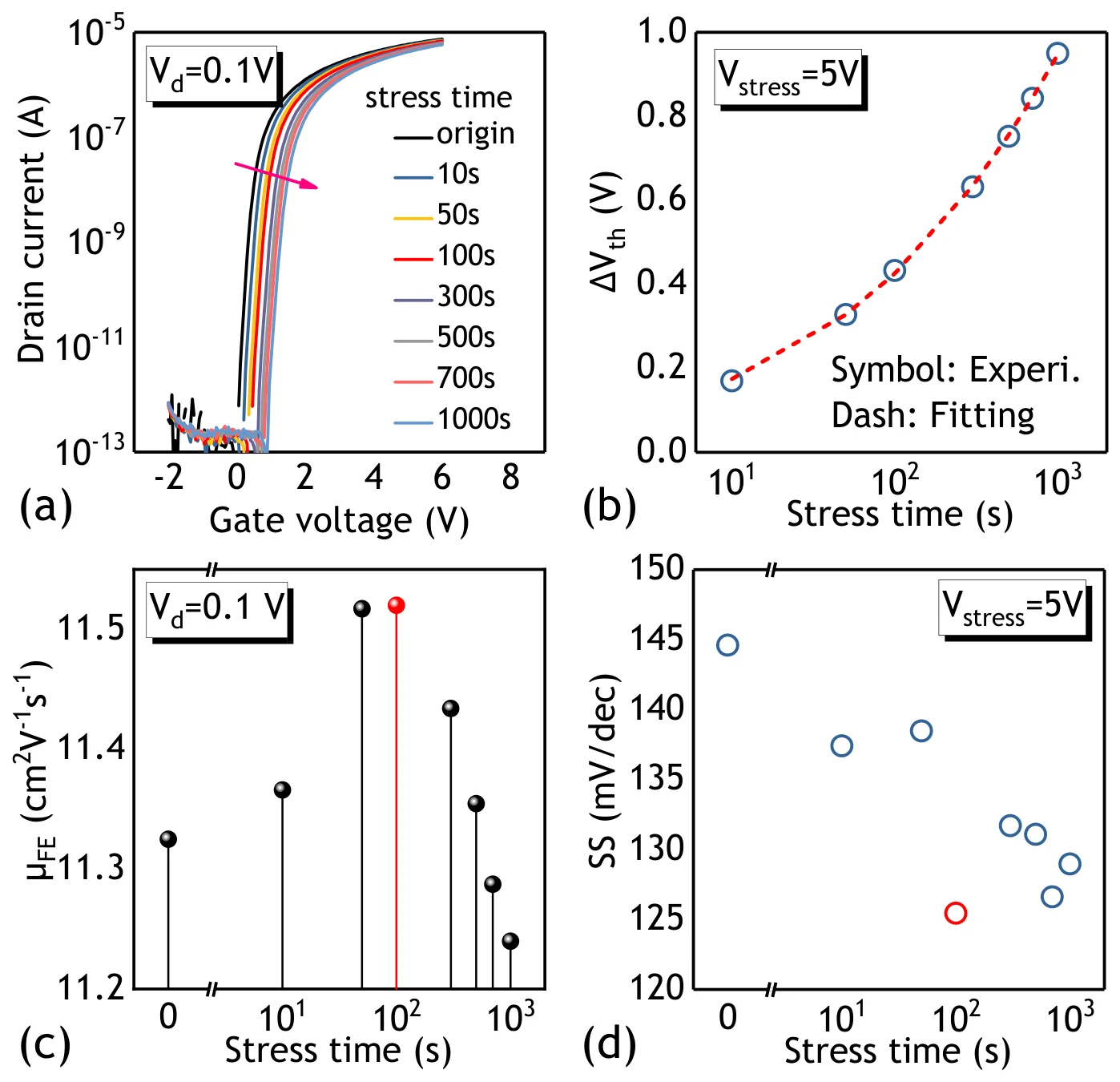
(**a**) Time evolution of *I*_d_–*V*_g_ characteristics of unpassivated a-IGZO TFT under *V*_stress_ = +5 V. The extracted (**b**) Δ*V*_th_, (**c**) *μ*_FE_, and (**d**) *SS* versus stress time of the device.

Figure 2a shows the temporal evolution of the transfer curves for the unpassivated a-IGZO TFT undergoing PBS at the *V*_stress_ of +5 V. The curves consistently shift in a positive direction over the entire process. This observed trend is associated with electron trapping phenomena within the gate insulator and/or at the channel interface induced by bias stress [12]. The extracted Δ*V*_th_ as the function of stress time is presented in Figure 2b, exhibiting a well-matched correlation with the stretched-exponential equation as defined [12]

(1) $$\Delta V_{\mathrm{th}}=\Delta V_{\mathrm{th}0}\left\{1-\exp\left[-{\left(t/\tau \right)}^{\beta }\right]\right\}$$

where Δ*V*_th0_ is the Δ*V*_th_ at infinite time, and *β* is the stretched-exponential exponent. *τ* is the characteristic trapping time, associated with an effective energy barrier that electrons in the channel must surmount before accessing the charge trap sites. By fitting the experiment data, the *β* value of 0.41 and *τ* value of 4.8 × 10^4^ s are derived, respectively. The extracted *β* is close to or even smaller than the literature results of a-IGZO TFTs featuring a SiO_2_ gate dielectric produced via PECVD [12], implying that the specimen has good interface quality between the Al_2_O_3_ and a-IGZO.

Figure 2c illustrates the time-dependent *μ*_FE_ of the unpassivated a-IGZO TFT against PBS at the *V*_ov_ of 2 V. Interestingly, *μ*_FE_ increases with stress time at the earlier part of PBS, peaking at 100 s. The abnormal behavior of mobility variation could be explained by the neutralization of the positively charged V_O_-related defects at the channel/dielectric interface, discussed in detail later on. Following this peak, the mobility gradually decreases with stress time. This degradation can be attributed to two coexisting mechanisms. Enhanced Coulomb scattering, induced by accumulated trapped electrons at the interface, impedes carrier transport as the primarily mechanism. In addition, deep-level defects created under PBS, predominately V_O_-related states in the a-IGZO bulk and at the dielectric/channel interface, can capture free carriers and reduce the effective channel carrier concentration, thereby further degrading the field-effect mobility. The role of these deep-level traps will be further confirmed by the density of states analysis presented later in this work. The subthreshold swing (*SS*) versus stress time for the unpassivated a-IGZO TFT is shown in Figure 2d. *SS* is defined as

(2) $$SS={\left[\frac{d(\log I_{d})}{dV_{g}}\right]}^{-1}=\log\frac{kT}{q}\cdot \frac{C_{ox}+C_{d}+C_{i}}{C_{ox}}$$

where *C_ox_* is the unit capacitance of the gate dielectric, *C_d_* is the unit capacitance of the depletion layer in the channel, and C*_i_* is the unit capacitance of interface states between the channel and dielectric. Similarly, *SS* decreases and reaches the minimum at a stress time of 100 s. As indicated by Equation (2), the *SS* is related to the *C_i_*, which is determined by the interface states density (*D*_it_). The observed decrease in *SS* during the initial stage of bias stress (stress time < 100 s) confirms a reduction in the *D*_it_, which means the interface quality is improved. The temporal evolution of SS exhibits the same characteristic time constant (~100 s) as that of *μ*_FE_ and the saturation current. This correlation suggests that the reduction in interface trap density is accompanied by a decrease in positively charged scattering centers at the interface, which is a prerequisite for the mobility enhancement in the early PBS stage. The consistency among *SS*, *μ*_FE_, and *I*_d_ will be further supported by the LFN analysis below.

**Figure 3:**
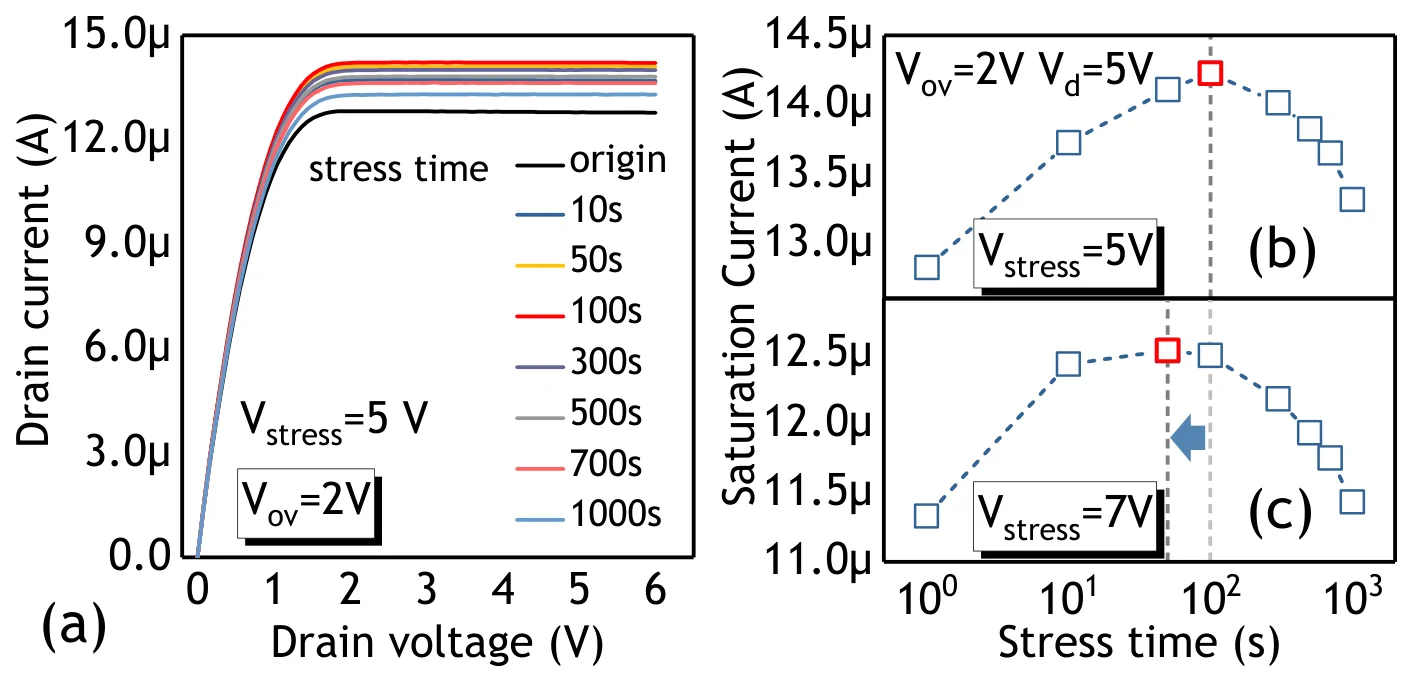
(**a**) Time evolution of *I*_d_–*V*_d_ characteristics of a-IGZO TFT without passivation at the *V*_ov_ of 2 V under *V*_stress_ of 5 V. The saturation current at *V*_ov_ of 2 V and *V*_d_ of 5 V with *V*_stress_ of (**b**) 5 V and (**c**) 7 V.

*I*_d_ versus stress time at the *V*_ov_ of 2 V under *V*_stress_ of +5 V is monitored, as shown in Figure 3a. Current in the linear region remains consistent across conditions. Differently, disparities occur in the saturation region. Figure 3b gives the stress time-dependent saturation current at *V*_d_ of 5 V, with the *V*_stress_ of +5 V. The current is gradually elevated under PBS at times less than 100 s and then declines with the stress time, corresponding to the mobility variation observed in Figure 2c. At a low *V*_d_, the lateral electric field is small in the channel, facilitating uniform electron flow from source to drain with minimal scattering by charged interface traps, thereby maintaining constant current levels in the linear region. As *V*_d_ increases, the carriers are accelerated in the channel, making them more susceptible to both scattering by interface defects and capture by deep-level traps, the latter of which further reduces the effective carrier concentration and degrades the drive current in saturation. The observed variation in saturation current cannot be directly correlated with the linear region *μ*_FE_ extracted at *V*_d_ = 0.1 V [Figure 2c], because the saturation region mobility, which governs the saturation current, is more sensitive to interface scattering under a high lateral electric field. The same reduction in interface charge density that yields a moderate improvement in linear region mobility can therefore produce a larger enhancement in the saturation current, as shown in Figure 3. This explains why the linear region current remains nearly constant during PBS while the saturation current varies markedly.

To confirm the reproducibility of the observed phenomenon, PBS measurements were repeated on two additional devices with identical fabrication conditions but slightly different channel dimensions (W/L = 100/50 µm). The non-monotonic evolution of mobility and saturation current, peaking at 100 s followed by a gradual decrease, was consistently observed across all devices, confirming that the reported trend is intrinsic to the PBS process rather than an artifact of a single device measurement.

**Figure 4:**
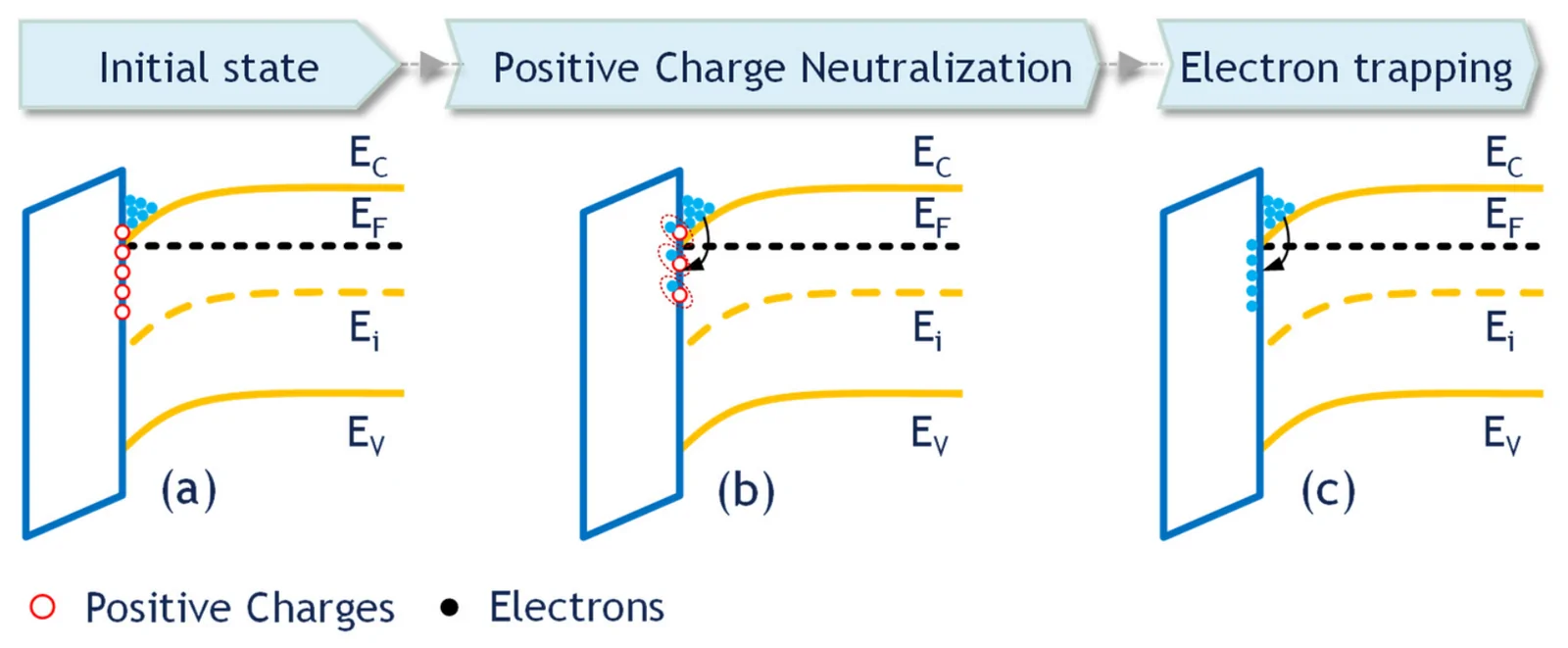
Energy band diagram of a-IGZO at different periods of PBS including (**a**) initial state before stressing, (**b**) at the period of positive charge neutralization, and (**c**) at the period of electron trapping.

The impact of PBS on a-IGZO TFT is delineated into three distinct stages, including the initial phase pre-stress, the positive charge neutralization stage, and the electron trapping stage, as depicted in Figure 4. The positively charged defects discussed here are considered to reside primarily at the Al_2_O_3_/IGZO interface and within the near-interface region of the a-IGZO channel, rather than in the Al_2_O_3_ bulk. This assignment is supported by the *SS* and LFN results, both of which are sensitive to interface-localized states. With this understanding, the impact of PBS on the a-IGZO TFT is delineated into three distinct stages. In the earlier period of PBS, inherent positively charged defects at the channel/dielectric interface are recombined by the electrons induced by PBS, resulting in an optimized interface quality [Figure 4a,b]. The improved interface makes the carriers transported in the channel endure less scattering, enhancing the mobility [Figure 2c and Figure 3b]. As the stress time continues to increase, positive charges are fully detrapped and then electron trapping is formed. The accumulated negative charges within the gate dielectric and/or at the channel interface increase carrier scattering, leading to mobility deterioration and a decline in output current [Figure 2c and Figure 3b]. Figure 3c shows the saturation current versus time under PBS with the *V*_stress_ of +7 V. Compared to the case where the *V*_stress_ is +5 V, the peak current is shifted to 50 s. With an increase in *V*_stress_, the heightened electric field (*E*) expedites electron capture at the channel interface, leading to reduced positive charge release time.

**Figure 5:**
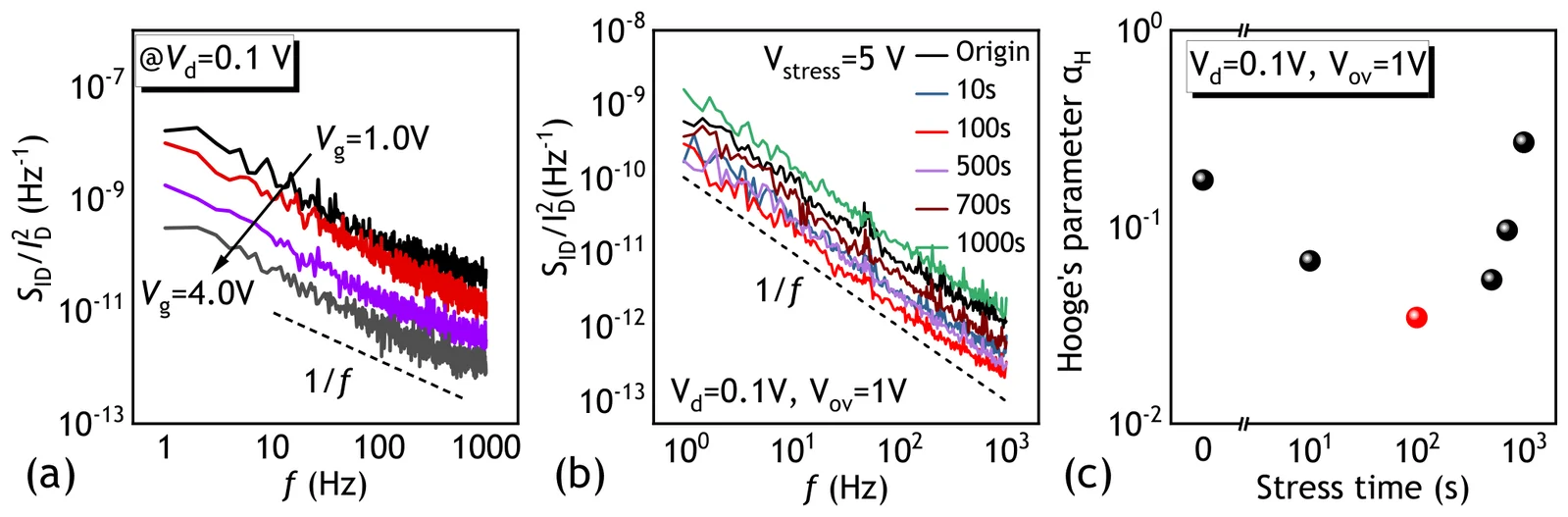
(**a**) Normalized noise power spectral densities (*S*_ID_/*I*^2^_D_) versus frequency of a-IGZO TFT measured at *V*_d_ = 0.1 V. (**b**) *S*_ID_/*I*^2^_D_ versus frequency *V*_d_ = 0.1 V and *V*_ov_ = 1 V before and after each node of PBS. (**c**) Extraction of Hooge’s parameter versus stress time at *V*_d_ = 0.1 V and *V*_ov_ = 1 V for a-IGZO TFT.

To further understand the underlying mechanism of PBS responses for a-IGZO TFTs, the LFN measurement was performed on the device. Figure 5a gives the normalized power spectral density (*S*_ID_/*I*_D_^2^) for the a-IGZO device measured at *V*_d_ = 0.1 V and *V*_g_ from 1 V to 4 V. All experimental fundings manifest standard 1/*f* characteristics and *S*_ID_/*I*_D_^2^ decreases monotonically with increasing *V*_g_. This trend is consistent with the Hooge mobility fluctuation model, which predicts that *S*_ID_/*I*_D_^2^ is proportional to 1/*N* (where *N* is the total number of carriers). The Hooge model is therefore adopted to describe the LFN behavior in this work. Figure 5b shows the *S*_ID_/*I*_D_^2^ for the a-IGZO device measured at *V*_d_ = 0.1 V and *V*_ov_ = 1 V in both pre- and post-PBS time nodes. Hooge’s Δ*μ* model [31] is described as

(3) $$S_{ID}/I_{D}^{2}=\frac{q}{C_{ox}\cdot W\cdot L}\times \frac{\alpha_{H}}{f}\times \frac{1}{V_{ov}}$$

where *α*_H_ is Hooge’s parameter. By experimental fitting, *α*_H_ values versus stress time are achieved and are shown in Figure 5c. A discernible pattern of reduced *α*_H_ levels is evident during stress durations below 100 s. It indicates effective alleviation of traps within the dielectric and/or at the dielectric/channel interface after positive charge neutralization in the PBS process, significantly diminishing sources of carrier scattering. Thus, current enhancement behavior occurs at the previous part of the stressing cycle [Figure 2c and Figure 3b]. At the stress time over 100 s, the *α*_H_ increases with stress time for the measured device [Figure 5b]. The rise in noise levels is owing to the new electron trapping after the positive charges were fully detrapped at the dielectric/channel interface. Hence, the output current at the same *V*_ov_ turns to decline as stress time increases further [Figure 3b].

**Figure 6:**
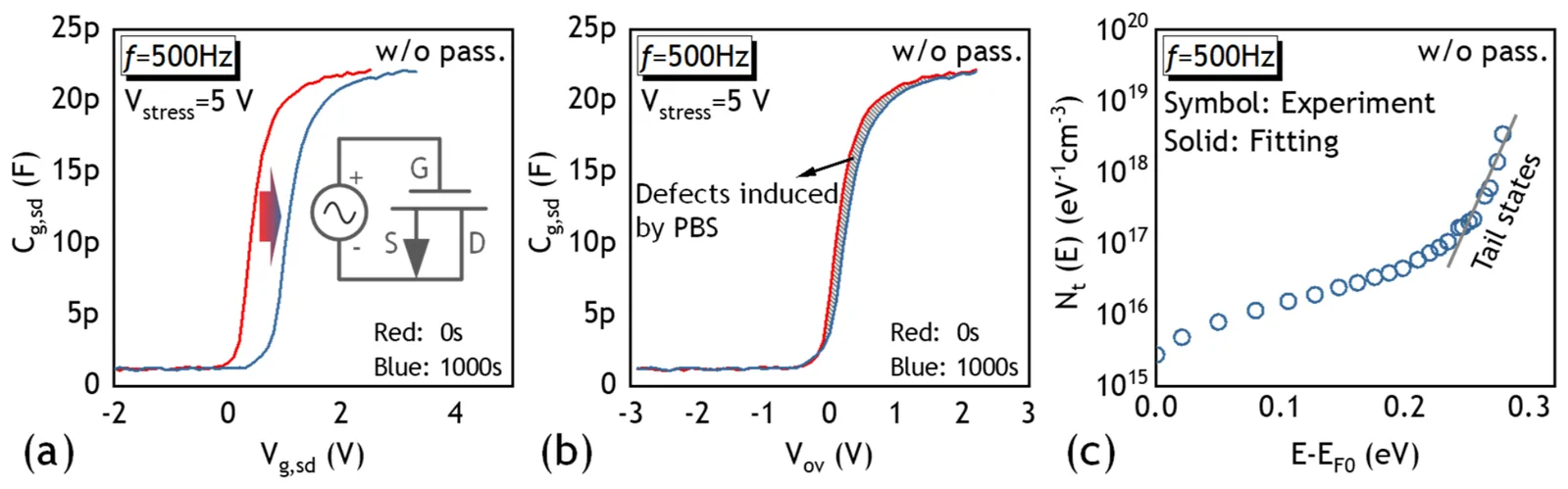
*C*_g,sd_ as function of (**a**) *V*_g, sd_ and (**b**) *V*_ov_ (*V*_ov_ = *V*_g,sd_ − *V*_FB_) for the TFT before and after PBS. (**c**) Extracted *N*_t_(E) against *E*-*E*_F0_.

To gain a deeper insight into the PBS responses of a-IGZO TFTs, capacitance measurements were carried out to assess the defects in a-IGZO caused by irradiation. The *C–V* measurements were conducted in a two-terminal configuration, where the gate electrode was linked to the high port, and the source and drain electrodes were shorted together and then connected to the low port of the LCR meter, as illustrated in the inset of Figure 6a. Figure 6a displays the output capacitance (*C*_g,sd_) of the device prior to and after PBS. As mentioned earlier, the positive shift in *C*_g,sd_ arises from negative charges trapped at the interface and/or within the bulk dielectric layers. To isolate the influence of PBS-induced defects in a-IGZO, the curves were aligned by shifting them according to the same *V*_ov_ (*V*_ov_ = *V*_g,sd_ − *V*_FB_), with *V*_FB_ being the flat-band voltage [Figure 6b]. This approach can remove the confounding effects of fixed charges and extra free carriers, allowing a direct comparison of the curves at a consistent energy level. After the two *C–V* curves are aligned, it is seen that the curves near *V*_FB_ nearly completely overlap, whereas a distinct discrepancy emerges in the vicinity of the strong accumulation region, as marked by the shaded area in Figure 6b. This discrepancy corresponds to PBS-induced defect states inside the bandgap of a-IGZO, which lower the gate-induced charge density under the same *V*_ov_, resulting in a reduction in output capacitance. For the fresh device, the *V*_g,sd_ (*C*_f_) can be given by

(4) $$\frac{1}{C_{f}}=\frac{1}{C_{ox}}+\frac{1}{C_{f,b}\left(V_{gs}\right)}$$

where *C*_ox_ is the capacitance of the gate dielectric, and *C*_f,b_ is the capacitance due to localized trapped charge in a-IGZO for the fresh device. After PBS, the *C*_g,sd_ (*C*_p_) can be depicted as

(5) $$\frac{1}{C_{p}}=\frac{1}{C_{ox}}+\frac{1}{C_{p,b}\left(V_{gs}\right)}=\frac{1}{C_{ox}}+\frac{1}{C_{f,b}(V_{gs})-C_{t,IGZO}(V_{gs})}$$

where *C*_p,b_ is the capacitance attributed to localized trapped charge in a-IGZO following PBS, which is made up of *C*_f,b_ and *C*_t,IGZO_. *C*_t,IGZO_ denotes the capacitance due to PBS-induced localized trapped charge in a-IGZO. Combining Equations (4) and (5), *C*_t,IGZO_ can be written as

(6) $$C_{t,IGZO}(V_{gs})=C_{ox}\left(\frac{C_{f}}{C_{ox}-C_{f}}-\frac{C_{p}}{C_{ox}-C_{p}}\right)$$

On the other hand, *C*_t,IGZO_ can be obtained as a function of *V*_gs_ from [32]

(7) $$C_{t,IGZO}(V_{gs})=\frac{q\Delta N_{t}}{\Delta V_{gs}}$$

where *N*_t_ is the density of traps per unit energy level and per unit volume induced after PBS in a-IGZO. Thus, *N*_t_ can be calculated as [33]

(8) $$N_{t}=\frac{C_{t,IGZO}}{q^{2}}=\frac{C_{ox}}{q^{2}}\left(\frac{C_{f}}{C_{ox}-C_{f}}-\frac{C_{p}}{C_{ox}-C_{p}}\right)$$

The surface potential (*ϕ*_s_) is depicted as [34]

(9) $$\phi_{s}=\int_{V_{FB}}^{V_{gs}}\left(1-\frac{C_{g,sd}}{C_{ox}}\right)dV_{gs}=q\left(E_{F}-E_{F0}\right)$$

Thus, *N*_t_ as a function of *E*-*E*_F0_ is obtained, as illustrated in Figure 6c. A measurement frequency of 500 Hz was selected to ensure full activation of the majority of trap states for trap density extraction. The extracted distribution, with trap densities on the order of 10^18^ eV^−1^·cm^3^ in the tail region, is consistent with previously reported sub-gap density of states in a-IGZO TFTs [32,33], confirming the validity of our extraction procedure. *N*_t_ increases with Fermi level, showing a double-exponential distribution. The steeper slope and higher density near the conduction band (CB) tail point to the dominance of shallow-level traps, which are due to the incorporation of PBS-induced defects in interface and/or within the active layers. The observation of a non-negligible deep-level component in this distribution supports the synergistic mechanism discussed earlier, through which deep-level traps capture free carriers and reduce the effective channel carrier concentration, thereby further degrading the field-effect mobility in the later stage of PBS.

**Figure 7:**
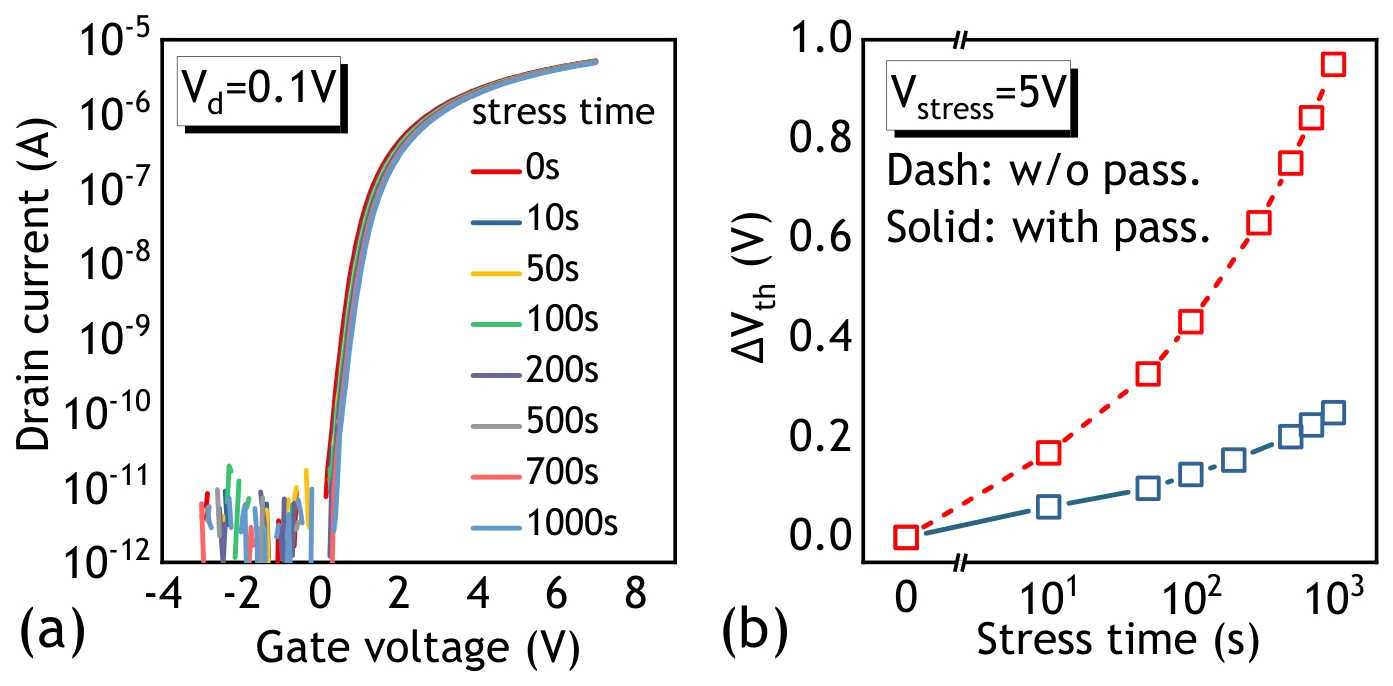
(**a**) Time evolution of *I*_d_–*V*_g_ characteristics of passivated a-IGZO TFT under *V*_stress_ = +5 V. (**b**) Extracted Δ*V*_th_ as function of stress time for the device with and without passivation, respectively.

Figure 7a shows the temporal evolution of the transfer curves for the passivated a-IGZO TFT under positive bias stress (PBS) at *V*_stress_ of +5 V. Throughout the entire stress period, the curves consistently shift in the positive direction, similar to the unpassivated device, which is attributed to electron trapping in the gate insulator and/or at the channel interface induced by bias stress [12]. Notably, the magnitude of the PBS-induced *V*_th_ shift is significantly reduced. As shown in Figure 7b, the PBS-induced Δ*V*_th_ of the a-IGZO TFT decreases from 0.97 V to 0.25 V at a stress time of 1000 s after passivation.

**Figure 8:**
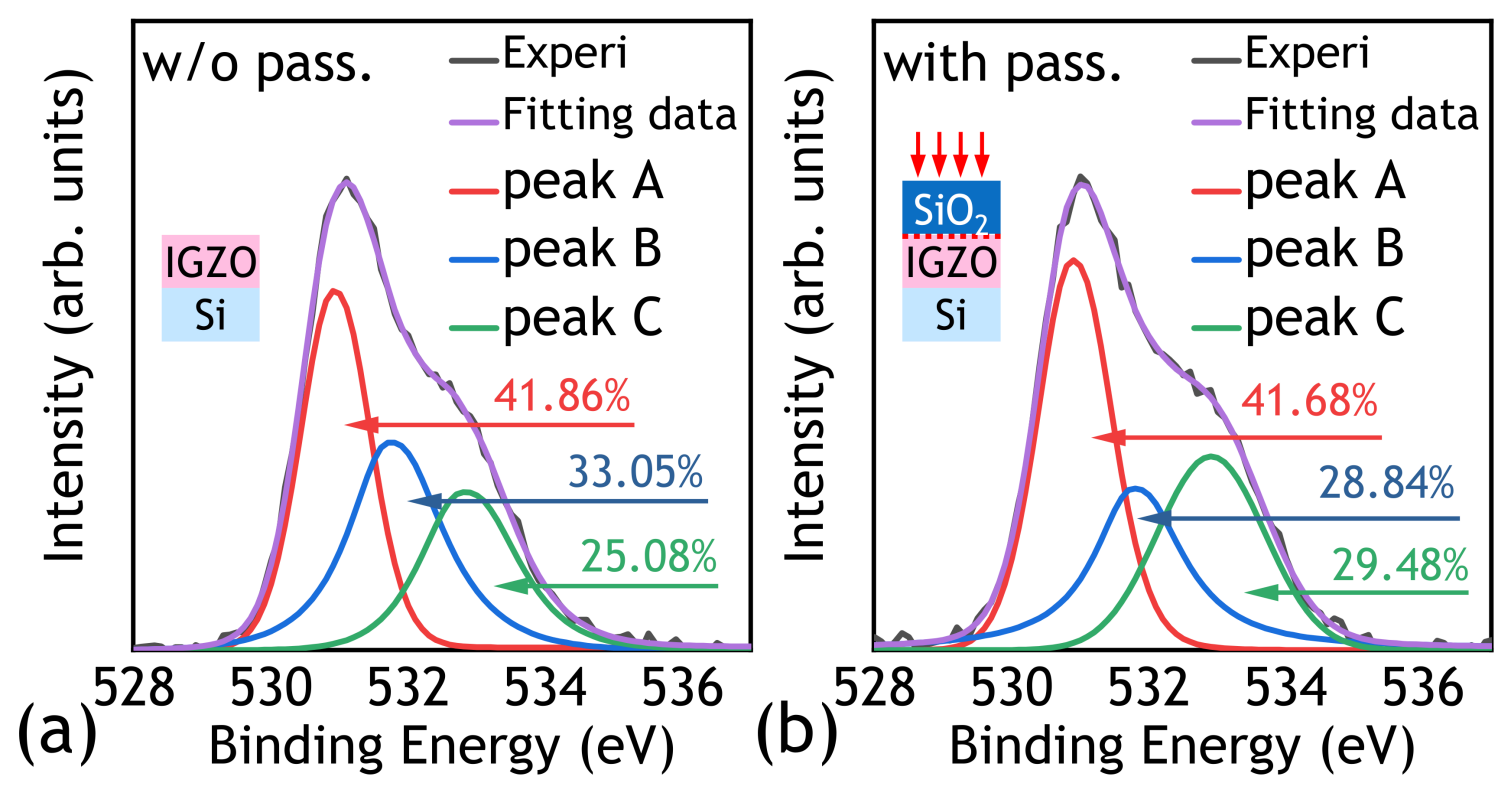
XPS O 1s spectra of the a-IGZO films without (**a**) passivation and (**b**) with SiO_2_ passivation.

Figure 8 shows the O 1s X-ray photoelectron spectroscopy (XPS) spectra of the unpassivated and passivated a-IGZO films. For the passivated sample, the SiO_2_ capping layer was removed by Ar^+^ sputtering prior to measurement to expose the underlying a-IGZO surface. The O 1s spectra are deconvoluted into three peaks: peak A (M-O lattice oxygen), peak B (oxygen vacancies, V_O_), and peak C (hydroxyl groups or hydrogen-related species, -OH). After passivation, the oxygen vacancy fraction (peak B) decreases from 33.05% to 28.84%. Meanwhile, the fraction of hydrogen-related species (peak C) increases from 25.08% to 29.48%. Regarding the enhanced PBS stability in passivated a-IGZO TFTs, the performance improvement originates from the synergistic combination of physical barrier protection and chemical defect passivation. On one hand, the PECVD-deposited SiO_2_ capping layer serves as an effective physical barrier, preventing the adsorption of ambient moisture and oxygen onto the back-channel interface. This eliminates extrinsic charge trapping centers and significantly suppresses environment-induced electron trapping. On the other hand, hydrogen incorporation during the PECVD process facilitates chemical defect passivation [35]. By saturating V_O_ and sub-gap states within the a-IGZO layer, hydrogen-mediated passivation reduces the initial density of trap states, thereby mitigating the *V*_th_ instability [13,36]. This is corroborated by XPS analysis, which shows an increase in hydrogen-related species (from 25.08% to 29.48%) and a concurrent reduction in oxygen vacancies (from 33.05% to 28.84%) after passivation.

It is worth noting that the PECVD-deposited SiO_2_ passivation layer also involves an intrinsic N_2_O plasma treatment process. In the PECVD process, N_2_O plasma serves as an integral precursor step to ensure the formation of a stoichiometric and high-quality dielectric film. From a mechanistic perspective, oxygen radicals (O*) generated in the N_2_O plasma can effectively diffuse into the back-channel surface of the a-IGZO, passivating V_O_ through the reaction: V_O_ + O*→ O_O_ [37,38]. This chemical passivation is evidenced by the improvement in the initial subthreshold swing (SS), supported by the reduced oxygen vacancy fraction observed in XPS. The highly reactive oxygen radicals generated in the plasma effectively passivate V_O_ at the back-channel surface, suppressing the formation of sub-oxide layers and reducing the density of shallow trap states. This plasma-induced surface repair, acting in synergy with the subsequent hydrogen-mediated defect passivation, significantly optimizes the electronic structure of the back-channel interface and contributes to the superior PBS reliability observed in this study.

**Figure 9:**
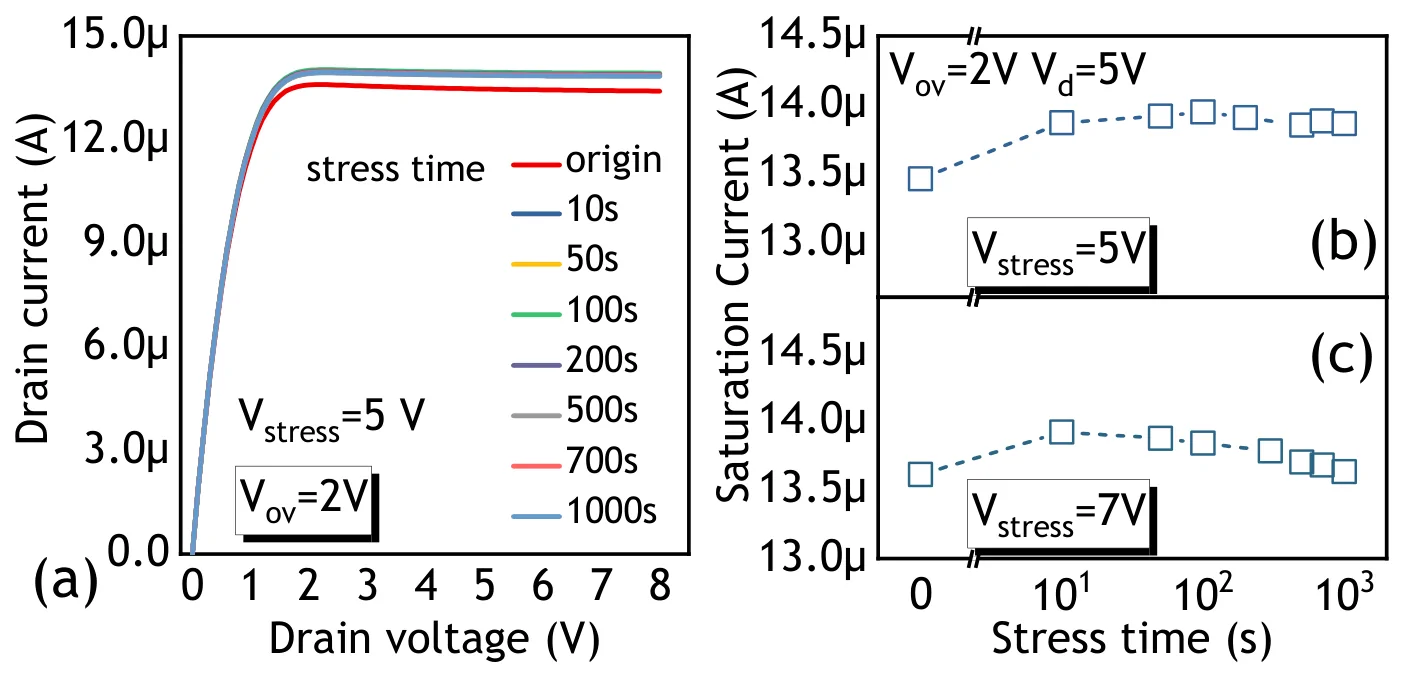
(**a**) Time evolution of *I*_d_–*V*_d_ characteristics of passivated a-IGZO TFT at the *V*_ov_ of 2 V under *V*_stress_ of 5 V. The saturation current at *V*_ov_ of 2 V and *V*_d_ of 5 V with *V*_stress_ of (**b**) 5 V and (**c**) 7 V.

*I*_d_ versus stress time at the *V*_ov_ of 2 V under *V*_stress_ of +5 V is monitored, as shown in Figure 9a. Current in the linear region remains consistent across conditions. It is worth noting that the contact resistance (*R*_SD_) remains stable throughout the passivation process. As evidenced by the consistent drain current in the linear region for both passivated and unpassivated devices, the Mo/a-IGZO contacts are not degraded by the PECVD passivation process, which is attributed to the moderate substrate temperature of 300 °C that preserves the chemical and structural integrity of the S/D contact interfaces. An analysis of the linear region transconductance (*g*_m_) at a low drain voltage (*V*_ds_ = 0.1 V) reveals that the *g*_m_ variation before and after passivation is less than 5%. Using the Y-function method, the estimated series resistance (*R*_sd_) accounts for less than 10% of the total channel resistance. Consequently, the distinct PBS-induced variations in the saturation region drive current observed in this study are primarily governed by the dynamics of carrier scattering and charge trapping at the dielectric/channel interface rather than by any contact-related degradation. This confirms that the passivation layer specifically targets the reduction in interface and bulk defects without introducing detrimental effects to the electrical contacts.

In the saturation region, a little current variation occurs during the bias stress process. Figure 9b gives the stress time-dependent saturation current at *V*_d_ of 5 V, with the *V*_stress_ of +5 V. Under PBS, the saturation current increases at the first stress time node of 10 s and then remains unchanged with increasing stress time. For the condition with *V*_stress_ of +7 V, as shown in Figure 9c, the saturation current increases at 10 s and subsequently decreases with stress time, exhibiting a similar trend to that of the unpassivated a-IGZO TFT. As previously discussed, passivation helps to reduce the interface defect distribution in a-IGZO TFTs. Therefore, for the passivated device, the saturation current reaches its maximum rapidly with increasing stress time. As stress time further increases, negative charge trapping occurs, leading to enhanced channel scattering and consequently reducing the saturation current under the same gate drive voltage. Nevertheless, for the passivated a-IGZO TFT, the PBS-induced variation in the saturation region drive current is very low, demonstrating an excellent passivation effect. Extended PBS measurements up to 3600 s confirm that the observed degradation trends for unpassivated devices and the stable performance for passivated devices persist beyond 1000 s.

## 4. Conclusions

The current enhancement behavior of a-IGZO TFT under PBS was explored in this article. *V*_th_ exhibits an upward trend with the stress time, which is raised from the capturing of electrons in the interface and/or bulk dielectric layers. The time dependence of Δ*V*_th_ aligns well with a stretched-exponential equation. In the initial state of PBS, the output current at the same *V*_ov_ elevates apparently with the stress time and reaches a peak at 100 s. The current boosting phenomenon is attributed to the positive charge neutralization occurring at the channel interface under PBS, resulting in reduced carrier scattering. The generation of electron trapping states after the full release of positive interface charge strengthens interface scattering, leading to a decrease in output current. Correspondingly, mobility increases first and then decreases during stress. The results of LFN are utilized to prove the proposed mechanism underlying PBS instability in a-IGZO TFTs. *C-V* characterization reveals that PBS-induced traps exhibit an exponential energy distribution, with shallow traps being predominant. Passivation greatly enhanced the PBS stability, attributed to hydrogen doping-induced defect passivation and the shielding of the back-channel interface from the air atmosphere.

## Data Availability

The experimental data presented in this manuscript were acquired through the following instruments and methods: electrical characterization was performed using an Agilent B1500 semiconductor parameter analyzer; capacitance–voltage measurements were carried out with an LCR meter (Keysight E4980); and chemical bonding states were analysed by X-ray photoelectron spectroscopy (XPS). All raw data were subsequently processed and analysed with custom MATLAB routines to extract the key device parameters and trap distributions reported in the work. The raw data are not publicly available due to proprietary and ongoing R&D constraints, but all essential results are reproducible from the manuscript and further details are available from the corresponding authors upon request.
